# Cell Sheet Transplantation for Esophageal Stricture Prevention after Endoscopic Submucosal Dissection in a Porcine Model

**DOI:** 10.1371/journal.pone.0148249

**Published:** 2016-03-01

**Authors:** Guillaume Perrod, Gabriel Rahmi, Laetitia Pidial, Sophie Camilleri, Alexandre Bellucci, Amaury Casanova, Thomas Viel, Bertrand Tavitian, Christophe Cellier, Olivier Clement

**Affiliations:** 1 Université Paris Descartes Sorbonne Paris cité, Assistance Publique-Hôpitaux de Paris, Department of Gastroenterology, Hôpital Européen Georges Pompidou, 20 rue Leblanc, 75015 Paris, France; 2 Université Paris Descartes Sorbonne Paris cité, Laboratoire imagerie de l’angiogenèse et plateforme d’imagerie du petit animal, UMR-S970, 56 rue Leblanc, 75015 Paris, France; 3 Université Paris Descartes Sorbonne Paris cité, Assistance Publique-Hôpitaux de Paris, Department of Pathology, Hôpital Européen Georges Pompidou, 20 rue Leblanc, 75015 Paris, France; 4 Université Paris Descartes Sorbonne Paris cité Assistance Publique-Hôpitaux de Paris, Department of Radiology, Hôpital Européen Georges Pompidou, 20 rue Leblanc, 75015 Paris, France; 5 Université Paris Descartes Sorbonne Paris cité, Laboratory of biosurgical research, UMR-U633, 56 rue Leblanc, 75015 Paris, France; EFS, FRANCE

## Abstract

**Background & Aims:**

Extended esophageal endoscopic submucosal dissection (ESD) is highly responsible for esophageal stricture. We conducted a comparative study in a porcine model to evaluate the effectiveness of adipose tissue-derived stromal cell (ADSC) double cell sheet transplantation.

**Methods:**

Twelve female pigs were treated with 5 cm long hemi-circumferential ESD and randomized in two groups. ADSC group (n = 6) received 4 double cell sheets of allogenic ADSC on a paper support membrane and control group (n = 6) received 4 paper support membranes. ADSC were labelled with PKH-67 fluorophore to allow probe-based confocal laser endomicroscopie (pCLE) monitoring. After 28 days follow-up, animals were sacrificed. At days 3, 14 and 28, endoscopic evaluation with pCLE and esophagography were performed.

**Results:**

One animal from the control group was excluded (anesthetic complication). Animals from ADSC group showed less frequent alimentary trouble (17% vs 80%; *P* = 0.08) and higher gain weight on day 28. pCLE demonstrated a compatible cell signal in 4 animals of the ADSC group at day 3. In ADSC group, endoscopy showed that 1 out of 6(17%) animals developed a severe esophageal stricture comparatively to 100% (5/5) in the control group; *P* = 0.015. Esophagography demonstrated a decreased degree of stricture in the ADSC group on day 14 (44% vs 81%; *P* = 0.017) and day 28 (46% vs 90%; *P* = 0.035). Histological analysis showed a decreased fibrosis development in the ADSC group, in terms of surface (9.7 vs 26.1 mm²; *P* = 0.017) and maximal depth (1.6 vs 3.2 mm; *P* = 0.052).

**Conclusion:**

In this model, transplantation of allogenic ADSC organized in double cell sheets after extended esophegeal ESD is strongly associated with a lower esophageal stricture’s rate.

## Introduction

Incidence of esophageal cancer is increasing throughout the world.[1] With recent improvements in endoscopic imaging, Barret esophagus with dysplasia and superficial esophageal neoplasia are more often diagnosed.[2,3] Their management has radically changed in recent years. Recent endoscopic treatments for superficial digestive tumors, such as endoscopic mucosal resection (EMR) or endoscopic submucosal dissection (ESD), has shown excellent results similar to surgical resection.[4,5] and offer lower morbid-mortality.[6] The Japanese Esophageal Association was the first to recommend EMR or ESD as the first line treatment for these lesions.[7] More recently, United States’ and European endoscopic societies have also approved EMR and ESD as the treatment of choice.[8] For extended lesions, ESD is preferred to EMR because it allows *en bloc* resection regardless of lesion size and shape.[4,9–11]

The major complication of large ESD is esophageal postoperative stricture formation. It appears to be correlated to the resection circumference. Currently, patients with ESD involving more than ¾ of the esophageal circumference develop strictures in up to 90% of cases. These strictures occur after two to four weeks and are responsible for feeding difficulties and deterioration of quality of life.[12] The wound healing process leading to stricture formation is only partially known. It starts with a local recruitment of inflammatory cells and fibroblasts, secondary responsible for the destruction of the different esophageal layer after excessive fibrotic formation. At the end of the process, complete re-epithelialization is usually obtained, but no regeneration of muscularis mucosae and a thinner inner muscle layer is observed. This phenomenon leads to ulcer constriction and consequently to esophageal stricture formation.[13]

Prevention of postoperative stricture formation is essential to extend ESD applications. Many works have been made in this direction with unsatisfactory results. In clinical practice, corticosteroids is the most widely used treatment but severe adverse event have been reported and thus, limited their application (severe infection, pleural effusion or osteonecrosis).[14,15] Okhi et al [16,17] have developed an innovative method of regenerative medicine with excellent results. They transplanted into the ulcer bed immediately after a large esophageal ESD, a single layer tissue engineered cell sheet of autologous oral mucosal epithelial cells. They initially developed this technique on a canine model and secondly, managed to transpose it to human beings. These results have yet to be confirmed.

Adipose tissue-derived stromal cells (ADSC) offer several advantages in regenerative medicine.[18–20] They are easily isolated,[21] are capable of paracrine activity (local immuno modulation, cell recruitment and neo vascularization) and are able to differentiate into different cell types (mesenchymal and non-mesenchymal lineage).[22] Moreover, ADSC are known to modulate keratinocyte-fibroblast interaction, improving the quality of regenerated tissue with the suppression of excessive fibrosis development.[23] Their application in different wound healing process has shown interesting results and they are currently being investigated in clinical trial in several fields (chronic inflammation, ischemic diseases).[24]

We conducted a comparative study to assess the effectiveness of a double cell sheet transplantation of allogenic ADSC to prevent stenosis after ESD in a porcine model.

## Materials and Methods

### Experimental protocol

The study was conducted from January to August 2014 at the Biosurgical Research Laboratory of the Alain Carpentier Foundation in Paris. All animals were treated according to the Standard Guidelines of the French Ministry of Agriculture and the experimental protocol received approval of the local ethics committee authorized for animal experimentations of the Paris Descartes University (registered number MESR 2035.02; Faculty of Medicine Paris Descartes, Paris, France).

Twelve 6 month-old female pigs (38–42 kg) from the same farm were used for our experiments. Pigs were randomized in 2 groups as follows: ADSC group received just after ESD 4 transplantations of a tissue engineered double cell sheets of allogenic ADSC placed on a paper support membrane; control group received 4 transplantations of a paper support membrane alone. All experimentations were performed under general anesthesia with continuous cardio-respiratory monitoring. Animals were prepared with a 24-hours solid food diet, premedicated with 10mg/kg of intramuscular ketamine and induced with 8mg/kg of intravenous propofol. An endotracheal tube was then inserted and anesthesia was maintained through isoflurane 2.5% inhalation.

### Esophageal endoscopic submucosal dissection

ESD was performed using a GIF-Q180 gastroscope (Olympus Optical Co, Ltd, Tokyo, Japan), a Videoscope System Exera II (Olympus Optical Co, Ltd, Tokyo, Japan) and an Electrosurgery unit ERBE ICC 350 (ERBE Technology, Germany). Starting 40 cm to 45 cm from the dental arch, hemi-circumferential dorsal marks were performed using a knife (Ovesco Technology, Tübingen, Germany) with soft coagulation. Separation of the mucosal layer from the muscular layer was obtained using submucosal injection of a glycerol solution containing indigocarmine dye. Incisions were performed externally to marks using endocut mode, and dissection was performed from the proximal incision to the distal incision using forced coagulation mode. At the end of the procedure, a hemi-circumferential 5 cm long scar exposing the muscular layer was obtained. All resected pieces were retrieved for morphological and histological analysis.

### Double ADSC-sheet construction

Adipose tissue-derived stromal cells were isolated by ABCell-bio society from the abdominal subcutaneous fat from a 6 month-old female pig sacrificed 1 month earlier. ADSC porcine status was confirmed by FACS showing cell expression of CD 90+, CD 105+ and CD 45-, CD 31- (ABCell-bio Society, Paris, France). Cells were cultured at 37°C and 5% CO2 with alpha minimum essential medium including 10% fetal bovine serum and 1% antibiotics (penicillin and streptomycin). For cells tracking after transplantation, they were labeled using the PKH-67 fluorophore, a phospholipids membrane cell marker compatible with the probe-based confocal laser endomicroscopy (pCLE) green probe (MINI 67-1KT, Sigma-Aldrich, Saint Louis, USA). The day before transplantation, PKH-labeled ADSC were harvested at early passage number (P4-P5) and seeded on a 12-well temperature responsive cell culture dish (Upcell Thermo Scientific, Fischer Scientific, France) prepared with the cell culture medium described above. Each well was coated by a poly-N-isopropylacrilamide membrane (at the bottom of each well) and seeded with 1.5 10^6^ cells. After 12 hours incubation at 37°C in a humidified atmosphere of 5% carbon dioxide, confluent cell sheets were then detached by incubating the plate at room temperature for 30 minutes. When the incubation temperature was reduced to 20°C (room temperature) all of the cultured cells spontaneously detached from the dish surfaces as intact ADSC sheets, without the need for enzymatic treatments. Then, ADSC sheets were gently aspirated with a pipette and layered on top of another one to obtain a double layer construct positioned on a hydrophobic paper (1.5 cm diameter), used as a support membrane.

### Endoscopic transplantation

**Fig 1:**
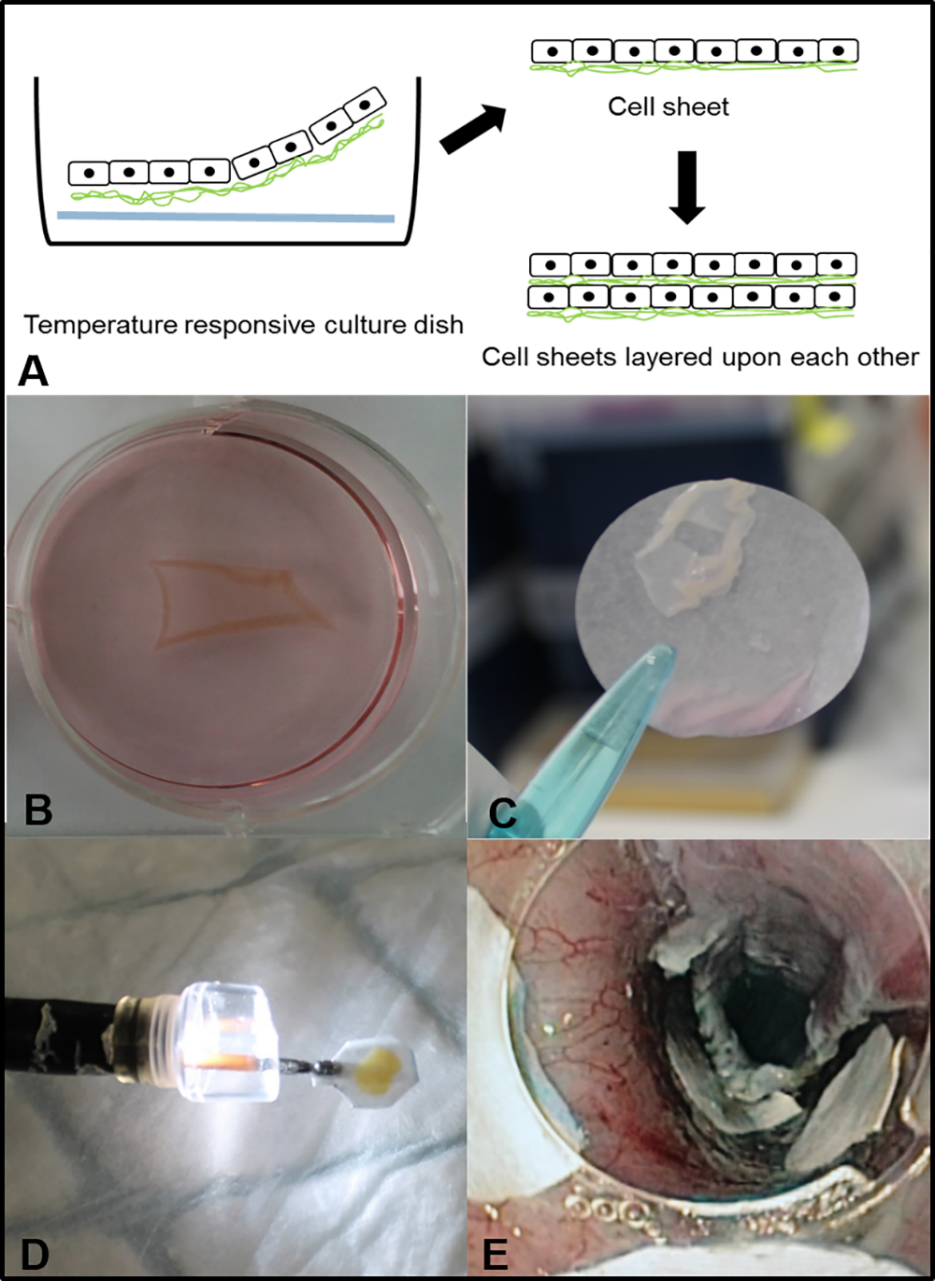
Transplantation procedure. A. Stem cell sheets were obtained by culturing PKH-labeled ADSC on commercial thermoresponsive culture dish. When the incubation temperature was reduced to 20°C (room temperature) all of the cultured cells spontaneously detached from the dish surfaces as intact ADSC sheets, without the need for enzymatic treatments. B. ADSC sheet completely detached from the dish surface. C. ADSC sheet harvested before graft construction using a transfer membrane. D. Protection of the graft under a large endoscopic cap before transplantation. E. Transplantation was allowed after a gentle graft application of ten minute using the endoscopic cap.

Immediately after ESD each animal in the grafted group received 4 double ADSC-sheets using a paper support membrane. This paper support membrane was grasped by endoscopic forceps, protected during introduction of the endoscope and transportation to the wound site by a large transparent endoscopic cap (Olympus Optical Co, Ltd, Tokyo, Japan) and then placed directly into the ulcer site. After 10 minutes of gentle application, the paper support membrane was released, resulting in a completely adherent support membrane on the ulcer bed (Fig 1). In the control group, each animal received 4 paper support membranes without cells using the same technique.

### Post-operative evaluation and follow up

#### Animal management

For each animal, after ESD analgesia was ensured using an intramuscular injection of 5mg morphine. They were treated with a 7 days antibiotic prophylaxis by amoxicillin 1g/day (Clamoxyl, GSK, Brendford, UK) and with a 28 days anti acid treatment by esomeprazole 40mg/day (Biogaran, Paris, France). After each procedure, liquids were authorized on day 1 and solids the following days. Animals were followed for 28 days and then sacrificed. On days 3, 14 and 28, we performed a multimodal evaluation of morphological stricture under general anesthesia (endoscopic, pCLE and radiologic evaluation)

#### Clinical evaluation

Animals were weighed on day 1 and day 28. A daily examination was performed according to the veterinary team’s assessments of pain, behavior, food intake, regurgitations and vomiting. Dysphagia was scored based on the Mellow-Pinkas score [25].

#### Endoscopic evaluation

Endoscopy allowed the description of the esophageal mucosal scar (inflammatory aspect: erythema, ulceration), the measurement of the stricture’s diameter using biopsy forceps and the possibility for the gastroscope to pass through the stenosis.

To evaluate the effectiveness of graft transplantation and assess a potential effect of the paper support membrane on the wound healing process, we defined the graft paper support detection rate (GPDR) as the detection of at least one paper support within esophageal scar. Concurrently, a pCLE evaluation was performed with a green laser probe, the minisonde Z (30 000 optics fibres 1.8mm diameter, 3.5 μm spatial resolution, de 100 to 170 μm working distance, 12 images/sec, Mauna Kea Technology, France). pCLE was used to research spontaneous signal of PKH-67 labelled transplanted cell sheet.

#### Radiological evaluation

Then after, radiologic evaluation (2 orthogonal incidences) was performed using baryte esophagography. The most relevant incidence was kept for analysis and we used the formula described below to measure the degree of stenosis.[26] A senior radiologist performed the analysis blindly from groups. Degree of stenosis was calculated as follow:

Degree of stenosis (%) = [1-(length of the short axis inside stenosis/length of the normal axis under stenosis) x 100)]

### Histological analysis

After sacrifice, esophagi were extracted and strictures areas were isolated for macroscopic evaluation and measurements. Esophagi pieces were then fixed in 10% buffered formalin, embedded in paraffin and processed into 4 μm-thick sections. Hematoxylin Eosin and Saffron (HES) was used to stain slides. Slides were digitalized for computerized analysis (Digitiser Hamamatsu Photonics ^®^, Massy, France) and analyzed blindly by a senior pathologist expert in digestive pathology (NDP.view software ^®^, Massy, France). Specific measurements were performed on the most pathological slide of each animal as follows: maximal thickness of the slide (extending from digestive lumen to serous layer), maximal thickness of re-epithelialization, maximal thickness of fibrosis, maximal surface of fibrosis, and maximal length of defect of *muscularis propriae* (characterized by the distance between the banks of *muscularis propriae* on each side). Fibrotic invasion of the inner and outer muscular layer was defined as the presence of fibrosis tissue continually from digestive lumen to muscular layers. Finally, inflammation analysis was performed semi quantitatively based on the detection of inflammatory cells through the different layers.

### Statistical analysis

Statistical analysis was performed using GraphPad Prism software (Graphpad software, La Jolla, CA, USA). Categorical data were expressed as percentages and compared with the Fischer exact test. Agostino and Pearson omnibus was used to confirm non-normality of our continuous data. We compared them with the Mann-Whitney test and expressed them as median values and range or mean ± standard deviation. A *P* value of less than 0.05 was considered to be significant.

## Results

### Procedure outcome

In the two groups, no complication was reported over ESD and transplantation procedure. The mean duration for esophageal ESD was similar between the two groups: 34.6 min vs. 31.5 min respectively for ADSC group and control group (*P*>0.05). Transplantation was successful for each animal, defined as the application of all four grafts in the esophageal bed scars. We harvested all resected specimen and size was comparable between the two groups. Average specimen height and width was respectively 2.6 cm and 1.3 cm; *P*>0.05 (S1 Fig).

### Clinical evaluation

We excluded one animal from the control group due to anesthetic complications. This animal was sacrificed on day 9 and was not replaced. Another animal from the control group showed one episode of spontaneously resolving hematemesis at day 25. No other serious complication was reported in our study. All clinical characteristics are presented in Table 1. Starting from day 11, we reported feeding troubles such as regurgitations and vomiting more frequently in the control group 80% (4/5) than in the ADSC group 17% (1/6); *P* = 0.08. Weight gain was -0,9 ± 3,6 kg in the control group and +0,7 ± 1,1 kg in the ADSC group; this difference was not significant.

**Table 1:** Clinical and endoscopical results on day 28.

Group	Pig	Weight variation (kg)	Day of diagnosis of dysphagia	Dysphagia score (*)	Mucosal description	Crossing through the stricture
**Control**						
	1	- 1	11	3	Ulceration	No
	2	0	13	2	Erythema	No
	3	2.5	17	1	Normal	No
	4	- 7	14	3	Ulceration	No
	5	1	14	2	Normal	No
**ADSC**						
	7	0.5	-	0	Normal	Yes
	8	0.5	-	0	Normal	Yes
	9	1	-	0	Normal	Yes
	10	0.5	-	0	Normal	Yes
	11	- 1	15	3	Ulceration	No
	12	2.5	-	0	Normal	Yes

^(^*^)^Based on Mellow-Pinkas reports, dysphagia was scored as follows:

0: normal swallowing

1: unable to swallow a proportion of the solid diet

2: able to swallow a semi solid diet

3: able to swallow liquids only

4: complete dysphagia including saliva

### Endoscopic evaluation

**Fig 2:**
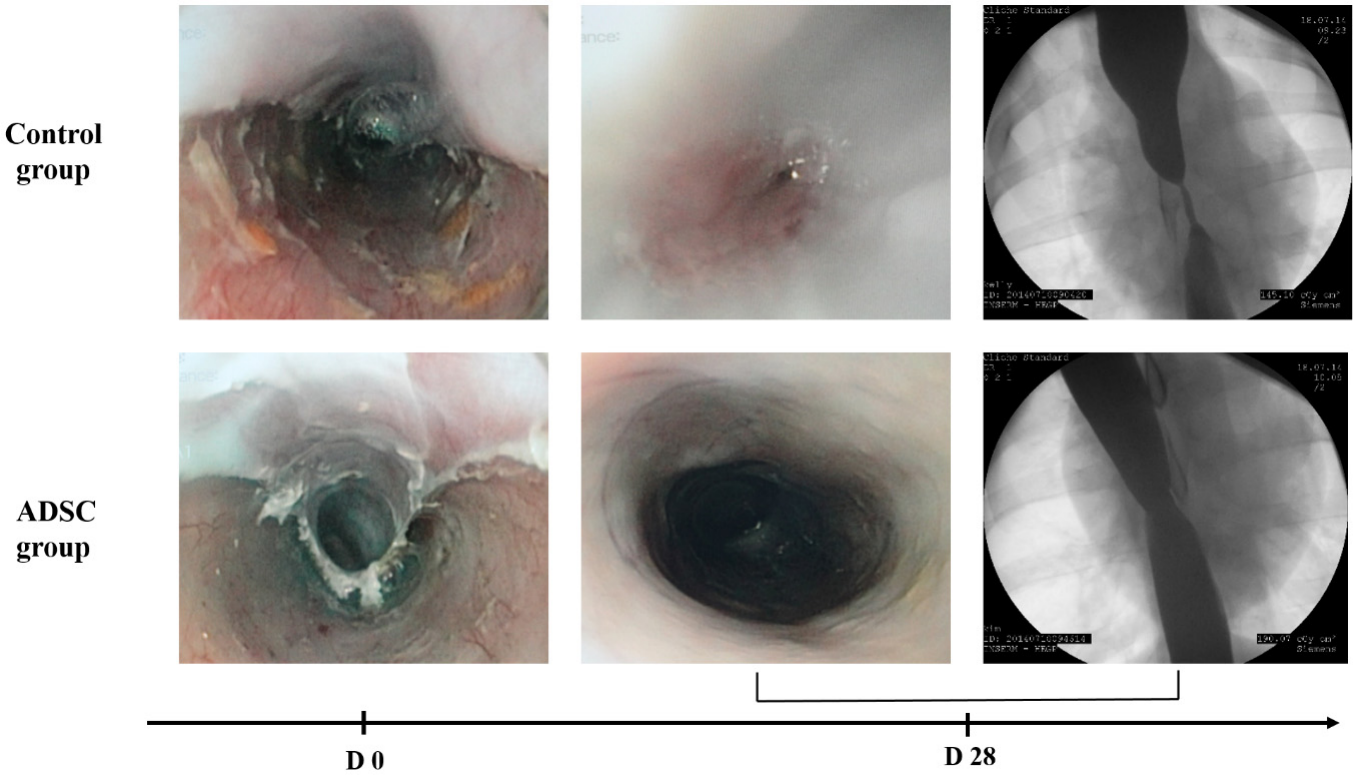
Endoscopic and radiologic images findings on day 28, in Control and ADSC group. Control group: inflammatory scar with persistent erythema narrowing a pinhole stricture, impossible to cross with the endoscope. In esophagography, presence of a long stringy stricture with upstream dilatation. ADSC group: complete scar re-epithelialization with inferior stricture allowing endoscope crossing. In esophagography, presence of a short moderate stricture without upstream dilatation.

On day 3, we identified no esophageal stricture and scar was completely covered with an inflammatory white coat. The GPDR was respectively of 66% (4/6) in the ADSC group vs 40% (2/5) in the control group; *P* = 0.56. Endoscopic evaluation is reported in Table 1 and typical findings are presented in Fig 2. One animal from the ADSC group presented a severe stenosis, resulting in inflammatory pinhole orifice that was impossible to cross with the gastroscope. Remaining animals from the ADSC group presented no severe stricture, allowing gastroscopic crossing of the stricture without palpable sensation. On the other hand, all animals from the control group presented severe strictures including two very tight strictures with a pinhole orifice. Regarding scar description, we identified less inflammatory mucosal scar in the ADSC group comparatively to the control group, with a lower rate of erythema mucosa and micro-ulcerations.

### Radiological evaluation

**Fig 3:**
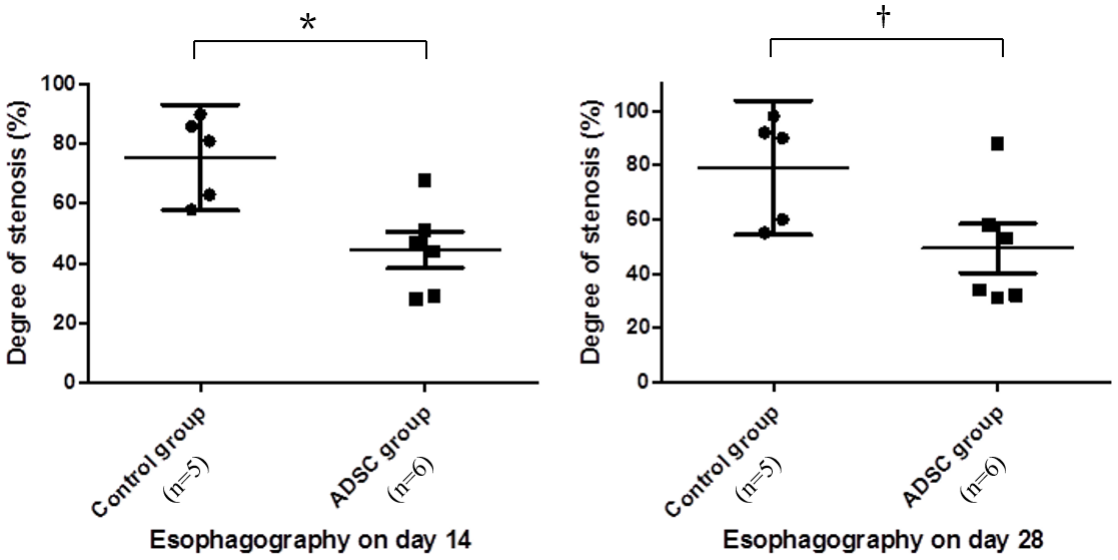
Radiological findings. Degree of stricture on day 14 and 28 was less important in ADSC group. Figures show the degree of stricture (%) on day 14 and day 28 with median and 95% IC. We used Mann-Whitney test to compare groups. (*) p = 0.017. (†) p = 0.03.

Degree of stricture at day 14 and day 28 was significantly less important in ADSC group in comparison with control group. Median esophageal diameter reduction was 44% vs 81% (*P* = 0.017) on day 14 and 46% vs. 90% (*P* = 0.03) on day 28, respectively for ADSC group and control group (Fig 3). All animals in control group and only one in ADSC group showed a severe stricture on esophagographies performed on day 28, with a stricture degree ranging from 55% to 98%. Typical stricture images of both groups are presented in Fig 2. The animal from the ADSC group that developed a severe stricture presented on esophagography a shorter stricture comparatively to animals from the control group.

### Confocal laser endomicroscopy evaluation

**Fig 4:**
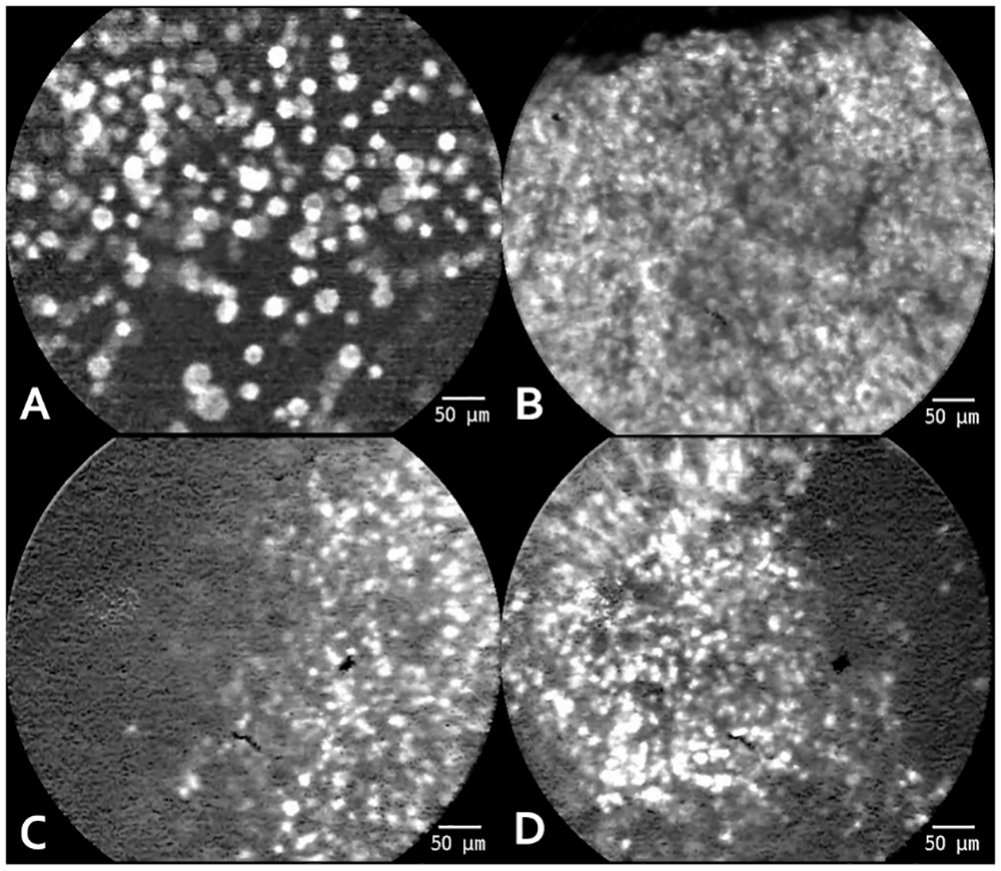
pCLE findings. pCLE on day 3 shows spontaneous and intense signal compatible with successful cell sheet transplantation. This signal was similar to those whom obtain *in vitro*. No signal was found at day 14 and 28 pCLE evaluation. A. ADSC visualized *in vitro* with pCLE, in suspension. B. ADSC visualized *in vitro* organized in cell sheet. C and D. ADSC visualiazed *in vivo* after successful cell sheet transplantation.

At day 3, four animals from the ADSC group showed in the treated area, a spontaneous, intense, punctuate and organized signal compatible with transplanted ADSC-sheet (Fig 4). Only one animal from the ADSC group presented the signal without support membrane identified within the esophageal scar (S1 Table) This signal was similar to those we obtained *in vitro*. Comparatively, no signal was observed in the control group. Regardless of group, pCLE evaluation performed at day 14 and day 28 did not retrieve this specific signal.

### Histological assessment

Macroscopically, we identified in the ADSC group 2 proximal strictures, one median stricture and 3 distal strictures. In the control group, we observed 3 proximal strictures and 2 distal strictures. Analysis of ESD resected pieces showed no difference between groups regarding sub mucosal depth resection and muscular layer achievement.

**Fig 5:**
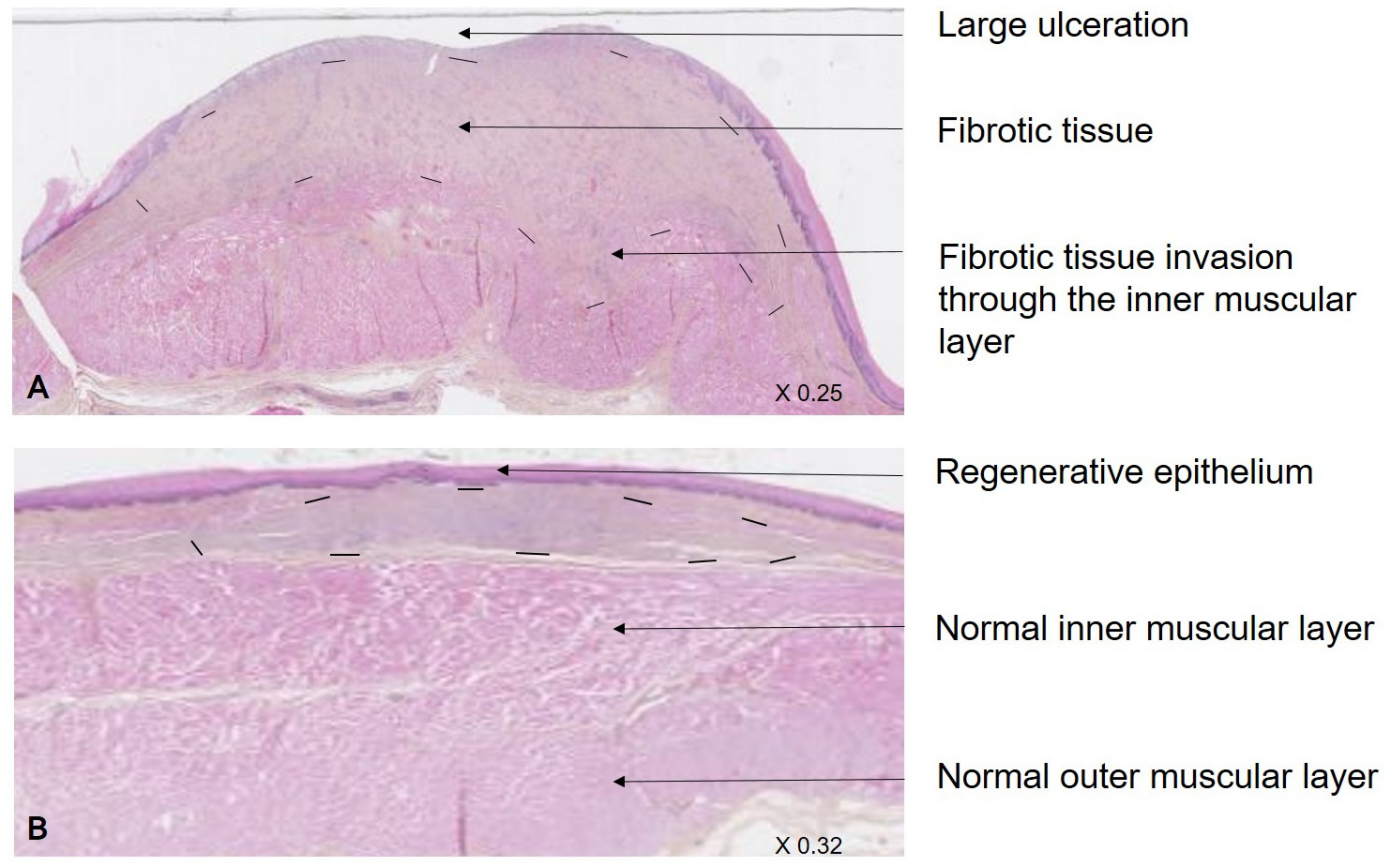
Histological assessment. HES labelled esophagus slides centered on stricture area, obtained after digitalization and read using NDP.view software (magnification x0.25 and x0.32). In control group (A), there was an intense development of fibrosis (delimited by dotted line), with a systematical large destruction of the muscularis mucosae, an invasion of the inner muscularis layer and a high re-epithelialization defect. Comparatively, in the ADSC group, healing process was improved with an increased scar re-epithelialization and a decreased fibrosis development, always respecting the inner muscularis layer.

All microscopically results are reported in Table 2. Histological analysis of normal oesphagi area was similar between groups (S2 Fig). In the control group, we observed an intense fibrotic tissue development, with a larger destruction of the *muscularis propriae* and a deeper fibrosis invasion through the inner and sometimes the outer muscularis layer (Fig 5). We also observed semi-quantitatively, an increased development of inflammation in the control group. As a consequence, the depth and the total surface of fibrosis were significantly higher in this group. Analysis of the epithelium thickness showed a trend for a thicker layer in the ADSC group comparatively to the control group.

**Table 2:** Histologic results on day 28.

	ADSC group (n = 6)	Control group (n = 5)	P-value (*)(†)
**Fibrosis thickness (mm) Mean ± SD**	1,6 ± 1,3	3,2 ± 0,7	0,052
**Fibrosis surface (mm²) Mean ± SD**	9,7 ± 8,3	26,1 ± 9,3	0,017
**Muscularis propriae defect (mm) Mean ± SD**	5,2 ± 4	9,4 ± 4,5	>0.05
**Epithelium thickness (mm) Mean ± SD**	0,14 ± 0,11	0,05 ± 0,07	0.09
**Inner muscularis layer invasion no. (%)**	2 (33%)	5 (100%)	0.06
**Outer muscularis layer invasion no. (%)**	0 (0%)	3 (60%)	0.06

^(^*^)^ We used Mann-Whitney test for continuous variable analysis.

^(†)^ We used Fisher test for categorical variables analysis.

## Discussion

Based on a porcine model, we showed the effectiveness of cell sheet transplantation for the prevention of postoperative esophageal stricture after large ESD. In our study, transplanted animals with allogenic ADSC sheets presented a lower degree of stricture and a lower fibrosis development in comparison with control animals. For the first time, monitoring of cell sheet transplantation was successfully performed with pCLE allowing an *in vivo* evaluation. Thus, we obtained a fluorescent signal compatible with the presence of transplanted cell sheet at day 3 in four animals from ADSC group.

Stricture formation after extended esophageal ESD remains challenging despite the use of medical preventive treatment such as oral or intravenous corticoids.[14,15] The promising field of regenerative medicine is working to restore structure and function of damaged tissues and organs, traditionally using isolated stem cell suspension or biodegradable scaffolds. The application of recent knowledge to the prevention of esophageal post-endoscopic stricture has drawn rising attention. After large esophageal resection, tubular scaffolds derived from porcine urinary models or porcine small intestinal submucosa combined with autologous oral mucosal epithelial cells were applied respectively in a porcine model and in a canine model.[27,28] The authors reported a decreased local inflammation and the induction of re-epithelialization from surrounding native epithelium. Amniotic membrane is known to present scaffold quality, promoting epithelial growth and allowing cell migration and proliferation. In a recent study, Barret et al evaluated the application of human amniotic after circumferential ESD in a porcine model. This application failed to prevent esophageal stricture, but resulted in a delayed stricture formation.[29] Others therapies such as direct injection of cell suspensions into the scar have been performed in different models. Honda et al. injected autologous ADSC in a canine model with a relative efficiency for stricture prevention.[30] They observed an accelerated process of mucosal healing and a diminution of mucosal constriction, with a mean degree of mucosal constriction of 45.3% in their injected group. In this model, authors evaluated stricture degree using macroscopic evaluation after longitudinal dissection of the esophagus. This method might be responsible for measurement bias. Direct cell injection is a minimally invasive method for cell transplantation. However, its major limitation is the poor cell viability (ranging from 1 to 32%), due to high mortality cell rate while the passage through the tip of the syringe.[31]In this approach, cell sheet constructs were chosen by virtue of their high cellular density and their ability to adhere to the native tissue. Hence, cells survival was improved into the site of transplantation. [32,33]

Human application of cell sheet construct has already been performed in several fields, such as corneal epithelium dysfonction, urothelium regeneration or skin defect.[34–36] ADSC are attributed to anti-inflammatory properties, local immune modulating effects, neovascularization induction and differentiation abilities into mesenchymal and non-mesenchymal lineage.[23,37] In our study, we used allogenic ADSC for tissue-engineered construction, organized in two layers of cell sheet for several reasons:

First, comparatively to other stem cell lineage, ADSC are easy to isolate in a large number and easy to expand *in vitro*. In our model, the use of allogenic cells rather than autologous cell was permitted because ADSC are also known to induce a high immunologic tolerance (absence of HLA-DR expression and modulation of T lymphocyte activity). [38,39]

Secondly, we assumed that local application of ADSC would decrease abnormal fibrosis and assist mucosal normal regeneration. We used a double-sheet construction in order to improve the effectiveness of transplantation. Two ADSC sheets instead of one sheet were chosen for the following reasons: 1/ an ADSC 3D construct made of two ADSC sheets layered up upon each other (with their preserved extracellular matrix) allow a better cell survival into the ulcer bed because it protects the cells from aggressive external environment; 2/ neoangiogenesis could be stimulated by the superposition of two ADSC sheets promoting proangiogenic factors diffusion, which enables engraftment; and 3/ a double ADSC sheet is more easy to manipulate during the endoscopic procedure. Two ADSC sheets instead of three or more sheets were chosen because the higher the number of sheets, the higher will be the risk of necrosis and cell death inside the 3D construct. Comparatively Okhi et al. applied a different type of cell sheet. They transplanted mature cell sheets approaching the native esophagus mucosal histological structure, composed of a stratified autologous oral mucosal sheet consisted of 3–5 layers. These sheets were obtained after 14 days of cell incubation using a temperature culture cell dish. Finally, based on previous studies we decided to transplant four double cell sheets per animal, with the objective to cover approximatively half of the wounded surface.

Our first hypothesis to explain the antifibrotic effect of ADSC sheet transplantation is the consequence of multiple factors released by the ADSC. This paracrine effect of ADSC seems to happen early, just after transplantation. Mechanisms of fibrosis are complex. After external aggression, as endoscopic resection, inflammatory response could be severe and uncontrolled resulting in abnormal healing with fibrosis development. ADSC probably influence this initial inflammatory response, particularly by producing TGFβ. [40,41] Moreover, with other factors as PDGF (platelet-derived growth factor), and IL13, TGFβ1 stimulates fibroblast differentiation into myofibriblast, thus improving healing process.[42]To monitor the effectiveness of cell sheet transplantation we used a cytoplasmic fluorescent contrast agent compatible with pCLE for cells labeling, which is an easy and accessible technique. This strategy had two main limitations: 1/ at D3, even though there was a signal, it gives no information on cells viability; 2/ the dilution of the signal after transplanted cells division could explain the lack of signal after day 3; and 3/ the PKH 67 fluorophore is known to present a decreasing signal over time.[43] Interestingly in the 4 animals of the ADSC group with a positive signal on day three, only 1 presented a signal without the persistence of a support membrane in situ. This result is an argument to confirm the success of ADSC sheet adhesion to the wounded area. As there were no specific makers of pigs ADSC, a possibility to highlight the cells on post sacrifice histology could be a stable fluorescent labelling. Thus Okhi et al. used a DNA-binding dye and a phospholipid-binding dye to evaluate the survival of transplanted epithelial cell sheets on ulcer wound beds after extended. They showed that eight days after transplantation, resected tissues showed the presence of epithelial cells covering most of the wound site. Kanai et al. also confirmed the success of epidermal cell sheet transplantation through immunostaining.[44] Another alternative is to use a stable fluorescent signal using cell transfection with, for example, the gene coding for the protein GFP (Green Fluorescent Protein) 509 nm wavelength which is compatible with pCLE examination. The major advantage of this technique is to know the cells viability. Nevertheless, our results are the first to confirm the success of cell sheet transplantation *in vivo*.

Despite the success of the transplantation procedure, one animal from the ADSC group developed a severe stricture with feeding difficulties. Confirmation of severe stricture was obtained on day 14 after morphological evaluation. Interestingly, endoscopic and radiological evaluation showed a proximal stringy stricture, but of very small dimension compared to strictures observed in the control group. Three hypothesis might explain this unfavorable evolution: 1/ a technical early failure of the cell grafts; 2/ a poor ADSC survival despite a positive fluorescent signal at day 3; and 3/ the cells destruction after day 3 that could be exacerbated by a difficult and longer pCLE procedure. Okhi et al [17] also reported a severe postoperative stricture in one patient with a quasi-circumferential 11 cm long esophageal ESD who received less oral mucosal cell sheets than initially planned.

One limitation of our study was the small number of animals included. Ideally, additional experiments could strengthen our results, especially for the clinical evaluation which was not different between groups. Even if clinical evaluation was very important, it was more difficult and less objective than endoscopic and radiological evaluation, in particular in terms of regurgitations and vomiting score in pigs. Concerning the weight gain, difference was not significant between the control group and the ADSC group. However, because of rapid growth in young pigs (6 month-old pigs), to reveal a significant weight gain difference seems to be challenging and would likely require a high number of supplementary animals.

In conclusion, double cell sheet transplantation of allogenic ADSC appears to be an effective preventive treatment of esophageal stricture after large endoscopic submucosal dissection. Moreover, pCLE seems to be a useful tool in order to monitor cells transplantation. Further studies are needed to confirm these results and explore the role of ADSC-sheets on the wound healing process. These preclinical results are very promising and encourage us to undertake the clinical evaluation of ADSC sheets, as a preventive treatment of esophageal stricture formation in patients treated by large ESD.

## Supporting Information

S1 Fig Analysis of the resected esophagus pieces after ESD. (TIF)

S2 Fig Histological analysis of the different layers of normal area of esophagi resected on day 28. (TIF)

S1 Table GPDR and pCLE findings on day 3. (DOCX)

## Abbreviations

ADSC: Adipose tissue Derived Stromal Cell
pCLE: probe-based confocal laser endomicroscopy
ESD: Endoscopic Submucosal Dissection
GPDR: Graft Paper Detection Rate
