# Supplementary figures and images for: Cell Sheet Transplantation for Esophageal Stricture Prevention after Endoscopic Submucosal Dissection in a Porcine Model
Source: PLoS One. 2016 Mar 1;11(3):e0148249. doi: 10.1371/journal.pone.0148249 (PMC4773126; doi:10.1371/journal.pone.0148249)

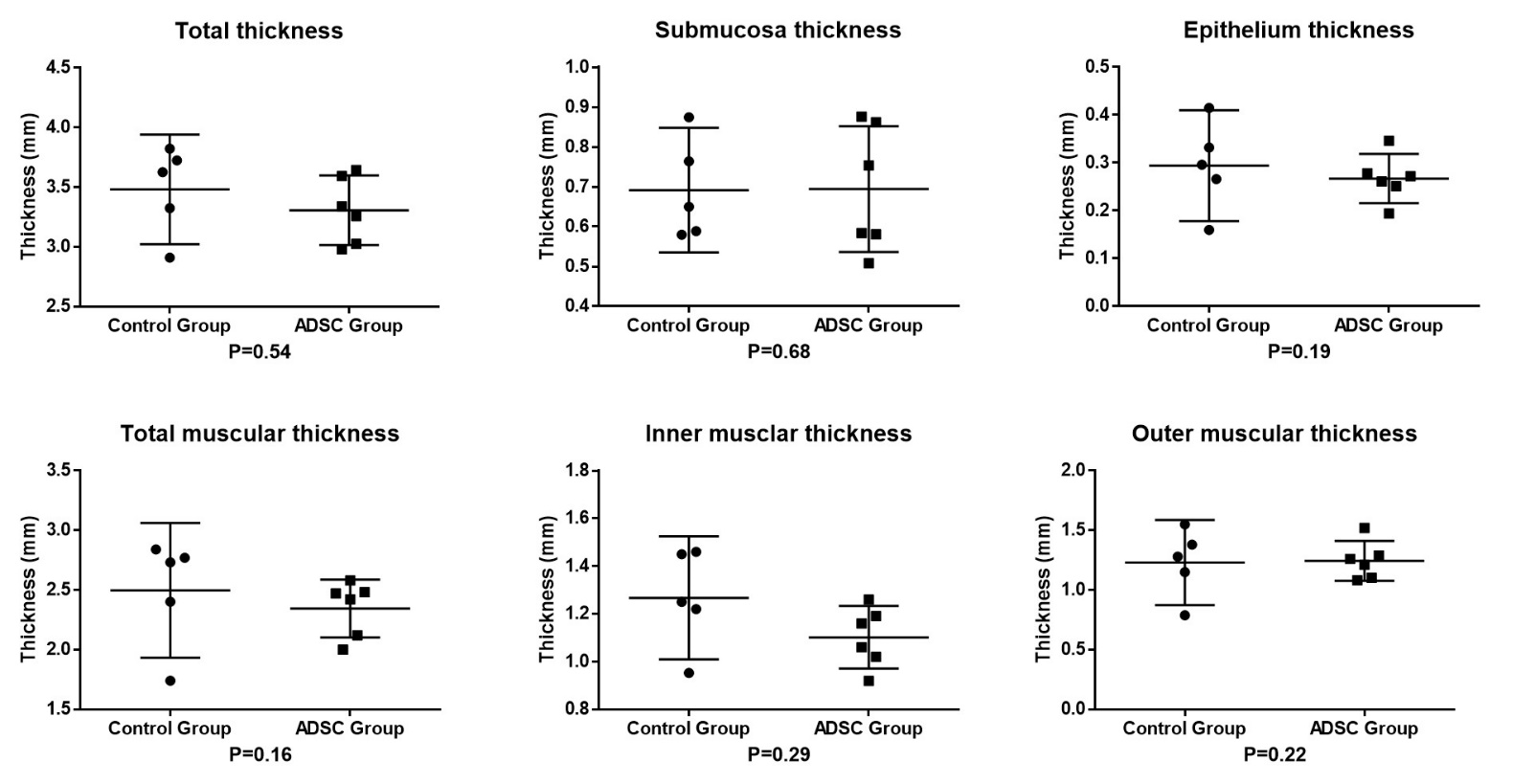

Supplement: S1 Fig — (TIF) [file pone.0148249.s001.tif]

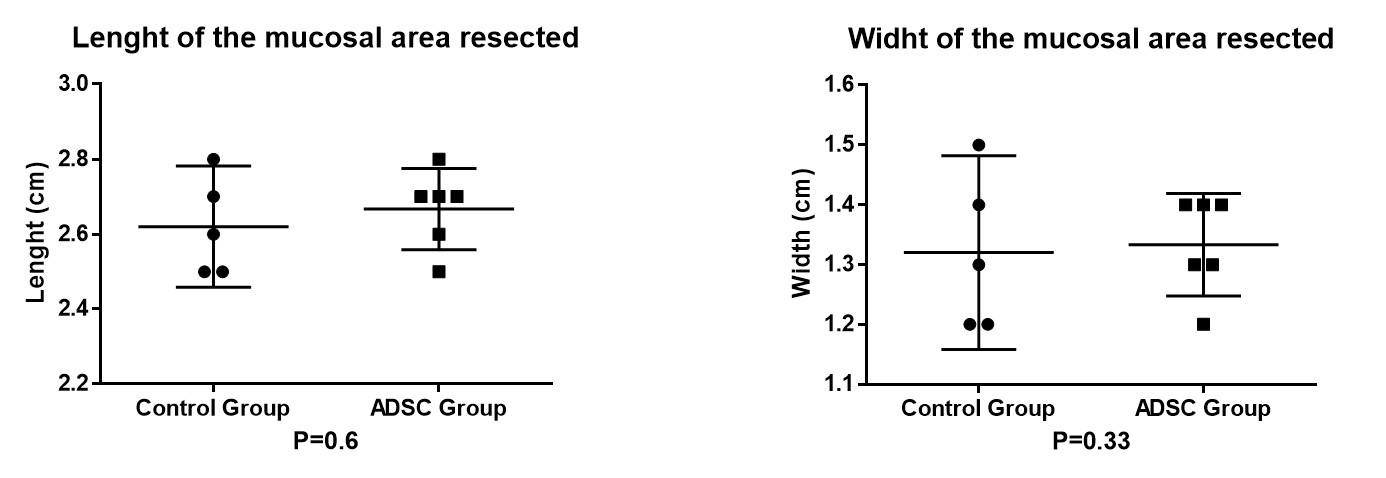

Supplement: S2 Fig — (TIF) [file pone.0148249.s002.tif]

**S1 Table:** *GPDR and pCLE findings on day 3*


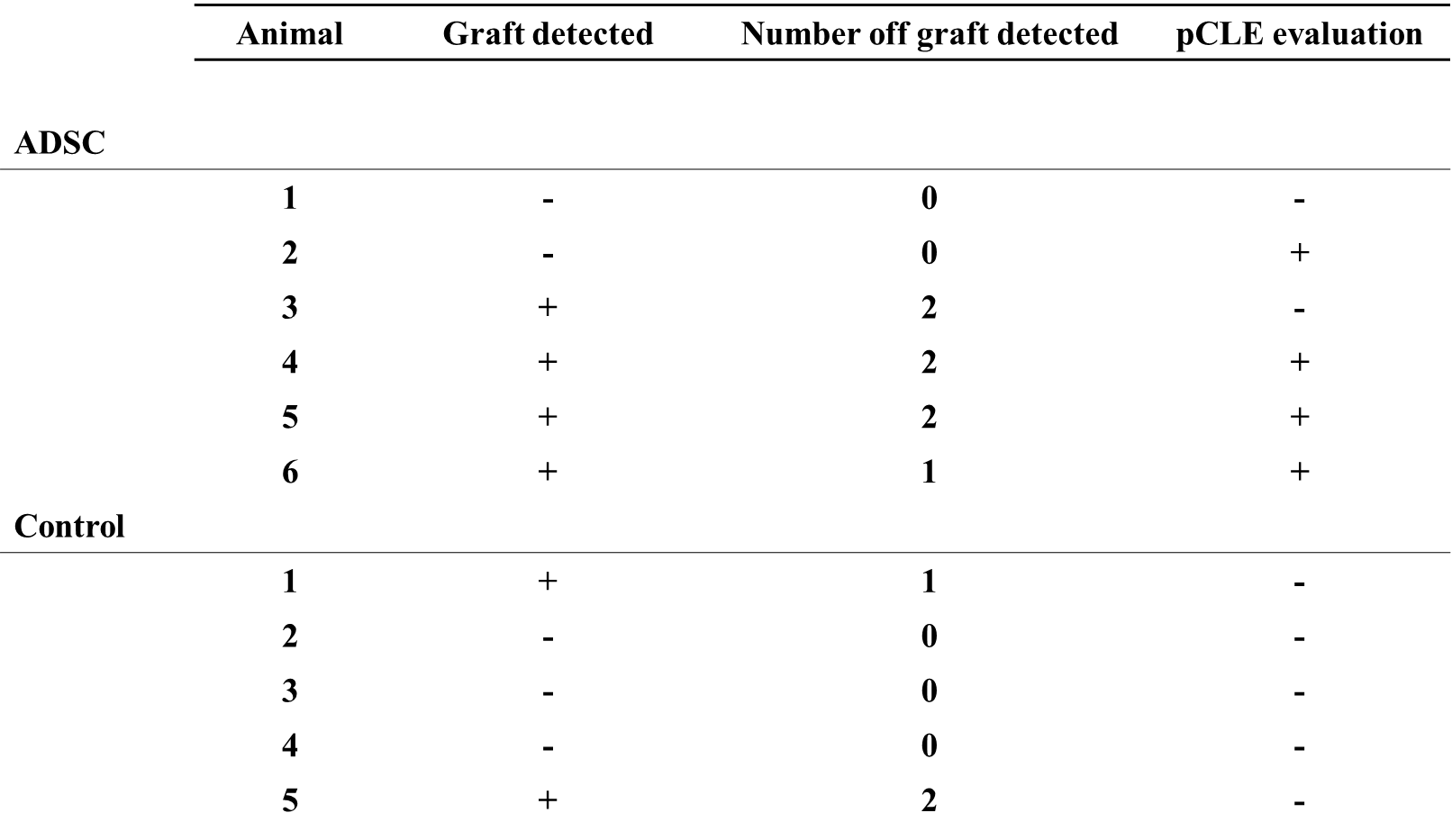

Supplement: S1 Table — (DOCX) [file pone.0148249.s003.docx]
